# Accelerating neuronal aging in *in vitro* model brain disorders: a focus on reactive oxygen species

**DOI:** 10.3389/fnagi.2014.00292

**Published:** 2014-10-27

**Authors:** Priscila Britto Campos, Bruna S. Paulsen, Stevens K. Rehen

**Affiliations:** ^1^Institute of Biomedical Sciences, Federal University of Rio de JaneiroRio de Janeiro, Brazil; ^2^D’Or Institute for Research and Education (IDOR)Rio de Janeiro, Brazil

**Keywords:** Parkinson, Alzheimer, schizophrenia, aged neurons, ROS, pluripotent stem cells

## Abstract

In this review, we discuss insights gained through the use of stem cell preparations regarding the modeling of neurological diseases, the need for aging neurons derived from pluripotent stem cells to further advance the study of late-onset adult neurological diseases, and the extent to which mechanisms linked to the mismanagement of reactive oxygen species (ROS). The context of these issues can be revealed using the three disease states of Parkinson’s (PD), Alzheimer’s (AD), and schizophrenia, as considerable insights have been gained into these conditions through the use of stem cells in terms of disease etiologies and the role of oxidative stress. The latter subject is a primary area of interest of our group. After discussing the molecular models of accelerated aging, we highlight the role of ROS for the three diseases explored here. Importantly, we do not seek to provide an extensive account of all genetic mutations for each of the three disorders discussed in this review, but we aim instead to provide a conceptual framework that could maximize the gains from merging the approaches of stem cell microsystems and the study of oxidative stress in disease in order to optimize therapeutics and determine new molecular targets against oxidative stress that spare stem cell proliferation and development.

## Introduction

In central nervous system (CNS) neurons, certain characteristic changes serve as cues for the advanced maturational state of both the cell and the organism. These include altered calcium metabolism, changes in cell energy metabolism secondary to altered cerebral blood flow, increased mitochondrial (mt) content and reduced oxidative metabolism (Stoll et al., 2011). In fact, at the cellular level, many age-related impairments ultimately converge to negatively impact cell respiration, which manifests as the overproduction of reactive oxygen species (ROS). It is for this reason that the roles of ROS in age-related change and in the exacerbation of age-related neurological diseases will be a primary focus of this review, although other indicators of cellular aging will also be mentioned. Considerable evidence exists to argue that the age-related overproduction of ROS is associated with either imbalances of cellular metabolism and the induction of apoptotic signaling pathway(s) that lead to cell death. One central objective of the current review is to explore and highlight specific relationships between metabolic and related molecular aspects of adult-onset neurological disorders, and the specific contributions of ROS overproduction to cellular damage of central neurons that cause the characteristic symptoms of these diseases.

### Differentiation and maturation of pluripotent stem cells

The introduction of techniques that produce induced pluripotent stem cells (iPSCs) was made by Yamanaka’s group in 2006, reportedly in response to difficulties in securing approval for work with human embryonic cells (Takahashi and Yamanaka, 2006). Their methods use human epithelial cells that are exposed to a series of characterized transcription factors under highly controlled experimental conditions. The end result is the production of a population of pluripotent cultured cells.

One important consideration regarding the use of stem cells for the investigation of adult-onset neuropathology is that cells after reprogramming events are restored to an embryonic state that renders them biochemically and functionally immature. This molecular and morphological immaturity also translates directly into a relatively heightened state of adaptive functional plasticity that could confer resilience to an experimental disease-onset state. Specific “youth-like” adaptive changes that are seen in iPSCs converted from fibroblasts are as follows: differentiation rates reminiscent of fetal cells (Mariani et al., 2012; Nicholas et al., 2014); upregulation of telomerase activity and elongation of telomeres (Marion et al., 2009); embryonic mt status with low levels of adenosine triphosphate (ATP; Suhr et al., 2010); reduced levels of mtDNA (Kelly et al., 2013); and increased mt membrane potential (Armstrong et al., 2010).

Many studies have been designed to develop new differentiation protocols, with the specific aim of producing different types of neurons *in vitro*. However, most of these protocols require long time periods to attain specific neuronal subtypes and frequently result in a low rate of cells with functional properties (Yan et al., 2005; Hu and Zhang, 2009). This issue has motivated researchers to overcome the expense and improbability of generating authentically chronologically “old” cells.

iPSCs became an interesting tool to model disorders as they retain genetic alterations from the donor. After being reprogrammed, these cells are usually differentiated into the desired cell type and can demonstrate a disease-related phenotype when compared to controls. However, as cells that are generated by most other differentiation protocols correspond to earlier developmental stages, recapping specific phenotypes that are developed by the patient becomes more difficult, especially in cases of late-onset disorders. In this review, we discuss some of the strategies that have been and are being developed in order to overcome this issue. We then describe some age-related phenotypes that are observed in late-onset disorders, which can be explored using this new strategy of patient-specific iPSCs.

### Accelerated aging of neurons

Stem cell research into adult disorders has been unlikely to flourish unless researchers could compress the developmental timeline. In order to reduce the extension of differentiation protocols and generate more homogeneous neuronal cells with robust synapse formation capabilities, Zhang et al. (2013) successfully forced the expression of a single transcription factor, neurogenin, in pluripotent stem cells; they were able to reach yields and purity levels of almost 100% within 2 weeks (Zhang et al., 2013). This strategy followed the same rationale that was used to induce cell reprogramming, resetting an entire biological system by inducing the overexpression of a few transcription factors. However, it remains unclear whether this approach prompts the cells to achieve an equivalent chronological age as those that are present *in vivo* in patients with age-related disorders.

In 2009, Saha and Jaenisch proposed that modeling long-latent disorders would be a challenge for the use of patient-iPSCs as the dynamics of disease progression in the patient was likely to be vastly different from any phenotype developed *in vitro* (Saha and Jaenisch, 2009). One suggestion made to overcome this issue was to accelerate the appearance of pathological phenotypes *in vitro* by exposing cells to environmental effects that may contribute to disease pathogenesis, such as oxidative stress.

In 2013, a major and possibly foremost advance in stem cell technology essentially accomplished this task with the groundbreaking development by the group of Lorenz Studer (Miller et al., 2013). They began their study with reconfirmed discrepancies in the molecular phenotypes between organically aged cells and corresponding iPSC pathway activities. Cells evaluated from elderly donors had age-related nucleic changes, protein loss, chromatin marker changes, and increased numbers of DNA mutations (Miller et al., 2013). Mitochondrial superoxide levels were also elevated compared with fibroblasts from younger counterparts, which incidentally reaffirmed the role of ROS mismanagement as both a potential cause and indicator of neural disorders. However, when these “aged” fibroblasts were used to generate iPSCs and subsequently differentiated, the resulting fibroblasts no longer showed the molecular signatures of being “aged” but more closely resembled the molecular status of cells developed from younger patients (Miller et al., 2013).

Recognizing the importance of an aged cellular state to the study of adult-onset diseases, these investigators isolated the genetic basis or underlying mechanism that compressed the physiological duration of aging cells. They developed a similar *in vitro* state as occurs for individuals with Hutchinson-Gilford progeria syndrome (HGPS). The central damaging mutation in this disorder is a lack of a cleavage site that leads to the accumulation of prelamin A in the nucleus and the consequent generation of progeroid syndromes (Smith et al., 2005). When this mutation was used in isolation at the experimental level, cells that carried the gene for accelerated aging and cells from phenotypic donors (both young and old donor categories) were compared for differences in cellular development via single cell indicators of physiological or molecular maturity. They discovered that carrying a mutation in lamin A (LMNA) was associated with the expression of molecular markers for premature aging. Miller et al. then developed an *in vitro* preparation that was useful for measuring the extent to which the transient expression of progerin was associated with this “aged” molecular profile. It was found that 3 days of progerin overexpression yields gene sets that are similar to that of accelerated aging for either young or old iPSC-derived fibroblasts. The following complement of aging-associated markers was observed: increased mt dysfunction, DNA damage, nuclear abnormalities, loss of heterochromatin, and reduced telomere length. Remarkably, they were able to identify degenerative phenotypes that related to PD after mimicking some aspects of normal aging in neurons, which demonstrated the utility of their approach for modeling late-onset disorders (Miller et al., 2013).

Controversially, cognitive functions are preserved in progeroid syndromes, which might be because LMNA is absent from the nuclear neural lamina (Jung et al., 2012). Recently, using iPSCs derived from patients with HGPS, Nissan et al. (2012) confirmed that the expression of a specific RNA commonly involved in neural development, miR-9, by neural cells potentially decreases the expression of LMNA (Nissan et al., 2012). The authors speculated that these findings could be related to the absence of cognitive impairments in children affected by progeria after neural cells lose progerin expression. Therefore, despite the overexpression of progerin triggering an aged-like state in neural cells derived from reprogrammed cells, it is not clear whether this pathway resembles the neural natural aging process. Consequently, it remains to be determined if the acquired phenotypes are enough to model other neurological late-onset disorders and whether they actually mimic the aging process in the nervous system.

### Candidate pathways to accelerate aging in neural cells

Developing physiologically complete models of accelerating neuronal aging may require the reconsideration of maturity and senescence being different events. The origins of neuronal senescence may belong outside the neuron, and possibly outside the brain. Unlike maturity, cellular senescence is represented by a stage where cells are stably arrested and also exhibit phenotypic changes (Rodier and Campisi, 2011). The global contributors to neuronal aging could also prompt homeostatic imbalances that influence repair mechanisms beyond recoverable limits, therefore initiating or exacerbating CNS diseases.

#### Insights from the aged brain

Searching for candidate genes that have the ability to propel neural development appears to be a reasonable approach, especially for studies of aging in this cell type. Neural cells are usually post-mitotic or quiescent, which means that they are not cycling frequently (Yoshikawa, 2000). As a result, the process of senescence does not necessarily follow the same rules and pathways as actively mitotic cells. The aging process in the brain is usually accompanied by an increase in the expression of p16^INK4a^ and a decrease in levels of FGF-2, VEGF, and IGF-1 (Shetty et al., 2005; Molofsky et al., 2006). There is also an aging-related decline in metabolic function and an increase in oxidative damage, which is followed by a reduction in the antioxidant capacity of the brain (Calabrese et al., 2007; Currais and Maher, 2013). In addition, other factors, such as epigenetic regulation and telomere erosion, are also related to the aging process, which contribute to the multifactorial nature of neuronal aging. The specific nature of how these factors contribute to the aging process has been reviewed elsewhere (Rafalski and Brunet, 2011; Yin et al., 2013) and not all of these aspects will be explored in this review.

Once acknowledged as a multifactorial event, it is a challenge to model the entire cellular aging process *in vitro*. However, as the overexpression of progerin can induce certain age-related phenotypes (Miller et al., 2013), it is reasonable to hypothesize that mimicking just one or several factors related to aging in neural cells would induce similar changes in neural cells. However, it is important to define whether these changes would be satisfactory to reproduce a specific phenotype related to late-onset disease. An alternative approach to reach this aim would be to find specific transcription factors or other regulators that could accelerate the aging process by influencing different parameters, such as epigenetic parameters and metabolic status, to produce the opposite effects of the reprogramming process.

#### Insights from other systems

##### Reactive oxygen species

ROS are oxygen-based radicals produced in most forms of eukaryotic cells through the activities of enzymes involved in the mt electron transport chain, cytochrome P450 activities, lipoxygenase or cyclooxygenase, NAD(P)H oxidases or uncoupled nitric oxide synthase (NOS), and peroxidases, amongst others (Nayernia et al., 2014). They are normal by-products of healthy cellular metabolic processes and are known to play physiologically useful roles in cell signaling; for example, as part of the immunity-oriented “oxidative burst” (Yang et al., 2013a). When cells, including neurons, are in a homeostatic balance, the availability of enzymes and scavenging molecules approximately matches local ROS production, preventing ROS accumulation and avoiding of cellular damage (Circu and Aw, 2010). The accumulation of ROS has been described as a potentially damaging by-product of aging and ischemia, inflammation, and neuropathology-related toxicities (Uttara et al., 2009; Liochev, 2013; Chaudhari et al., 2014).

Viteri et al. (2010) revealed using the HGPS model that oxidative abnormalities in fibroblasts play a large role in premature aging and that the nature of these changes were consistent with the normative patterns of cellular aging. More specifically, age-related increases in H_2_O_2_ production reduces FACE1 expression and thereby impacts levels of prelamin A (Ragnauth et al., 2010). The findings of Viteri et al. (2010) of typical changes of accelerated aging in progeroid models reaffirms the importance of understanding how ROS is implicated in cellular aging. In fact, the inappropriate management of the cellular redox status is a core theme in many theories that explain adult CNS aging and neuropathology (for review see: Hsieh and Yang, 2013; Kulak et al., 2013). The concept that ROS accumulation can be a major precipitating event in CNS pathology has been presented in many articles and reviews (Nunomura et al., 2001; Dias et al., 2013; Popa-Wagner et al., 2013). Moreover, unchecked, elevated levels of ROS eventually impair and oxidize DNA, proteins, lipids, and/or sugars, which creates cellular dysfunction that leads to apoptotic and non-apoptotic forms of cell death (Popa-Wagner et al., 2013).

On the other hand, Le Belle et al. demonstrated in 2011 that ROS levels were maintained in stem cells at relatively elevated levels compared with expected values from adult neural tissues; moreover, they argued that high levels of ROS were central to maintaining the proliferative state of these neural stem cells (Le Belle et al., 2011). The beneficial roles of high levels of ROS for this form of neurogenesis involve the phosphatidylinositol 3-kinase (PI3K)/Akt signaling cascade. Correspondingly, experimental reductions in cellular ROS levels have been associated with reduced rates of cell growth and proliferation for neural progenitor cells. Interestingly, their findings were generalized across both *in vitro* and *in vivo* conditions. Such a scenario may call for further investigation of the specific range of ROS that induces cell damage or the maintenance of cell culture conditions for neural progenitors and neurons with accelerated aging.

##### Adenosine

In the brain, adenosine forms part of a series of reactions that provide free energy for neuronal function; however, adenosine alone also serves as both a neurotransmitter and neuromodulator (Boison, 2008). Adenosine signaling is mediated through at least two adenosine receptors: A1 and A2a. The earliest indications that adenosine signaling may influence aspects of cellular aging and potentially initiate senescence came from investigations into cancer research and lymphocytes (Parish et al., 2013; Yang et al., 2013b). While there remains a void in the neuroscience literature regarding the candidacy of adenosine as an aging factor in neurons, certain animal studies support its inclusion in the current list of factors that are noteworthy of further consideration. For example, adenosine receptors in mouse increase in an age-dependent fashion, which suggests adenosine may be involved in the aging mammalian brain in general (Castillo et al., 2009).

Changes in enzymatic metabolism that occur with age appear to be a central event underlying adenosine imbalance, if not the most important factor. Aged rat neurons and human fibroblasts have been shown to exhibit significantly increased adenosine kinase activity (Liu et al., 1986; Mackiewicz et al., 2006) and decreases in adenosine deaminase levels (Ghneim et al., 2003). Additionally, ATP levels measured in cultures of aged fibroblasts are lower than equivalent values measured in samples of young fibroblasts (Miyoshi et al., 2006). Finally, aged samples are more prone to hydrogen peroxide-induced cell damage and a decline in mt function (Miyoshi et al., 2006). Taken together, this evidence is useful to suggest that adenosine accumulates in increasingly old brains due to modifications in enzymatic complexes that monitor and regulate energetic or redox states. That much of the work referenced in this section was successfully carried out in aged fibroblasts is suggestive of the suitability of cultures of age-accelerated neurons to determine how this signaling cascade accounts for physiological correlates of aging in CNS-specific neurons. More importantly, the particular combination of altered adenosine signaling and aged neurons upon a backdrop of a neurological disorder (such as neurons transformed from patients with schizophrenia) would become a powerful tool to discover which factors trigger these disorders.

### Additional challenges to the system

According to a second category of postulates regarding the biological causes of aging, the original sources of neuronal senescence could reside extraneuronally and potentially even outside the brain (Rodier and Campisi, 2011). To a large extent, recapitulations of the aging process in iPSCs have not emulated or addressed these potential triggers. Developing physiologically complete models of accelerated neuronal aging may require the reconsideration of this issue and the development of protocols that can emulate more global or organism-wide conditions of aging. Specifically, it is known that the aging brain is increasingly poorly served by age-related changes in oxygen or nutrient absorption, ion gradients, or facilitated ion transport, all of which depend on the integrity and health of cell membranes and transcriptional states. A practical example of the influences that circulating substances exert on the prevention of brain aging are recent studies that showed parabiosis connecting young and old mice. When the circulatory systems of young and old mice are connected, it is possible to observe reversion of some age-related aspects in the brains of the old mice (Katsimpardi et al., 2014; Villeda et al., 2014). Furthermore, the systemic administration of blood plasma samples from young mice into aged mice improved age-related cognitive impairments in terms of both contextual fear conditioning and spatial learning and memory (Villeda et al., 2014).

It is also important to consider that *in vivo*, local sources of ROS and biochemical cues of aging stem from both neuronal and non-neuronal sources. For example, it is known that LMNA production results from the processing of prelamin A by the removal of the farnesyl residue by FACE1, a metalloproteinase, in both neuronal and non-neuronal CNS cells (Liu and Zhou, 2008). Defects in this metalloproteinase such as the lack of a key cleavage site (as in case of progerin) leads to the accumulation of prelamin A in vascular smooth muscle (Ragnauth et al., 2010), which is important for controlling or “matching” blood flow to neuronal demand. Functional mismatches in delivery and neuronal metabolic demand may be further exaggerated by the increased rigidity and frailties of the aging cerebral vascular system, and thereby constitutes a source of oxidative stress (Ragnauth et al., 2010). Finally, another form of change coincident with truncated LMNA is compromise of the blood brain barrier, which could further exaggerate pre-existing biochemical vulnerabilities and/or the extracellular accumulations of ROS and consequent cellular damage.

Moreover, it is also known that chronic inflammation is associated with normal aging and age-related pathophysiologic processes and diseases, including AD, schizophrenia, and cancer, among others (Wyss-Coray, 2006; Federico et al., 2007; Saetre et al., 2007). The inflammatory process is frequently accompanied by the generation of free radicals and mediators, such as chemokines and cytokines, which converge on the production of reactive species (Federico et al., 2007). The “senescence-associated secretory phenotype” (SASP), which includes growth factors, proteases, chemokines, and cytokines, are usually induced by cellular senescence and act in an autocrine feedback loop to reinforce the senescence growth arrest and recruit components of the immune system to clear senescent cells. The first manifestation of senescence is the expression of IL-1α, a cytokine that binds to the IL-1 receptor to initiate the transcription of factors such as NF-κB and C/EBPβ. These transcription factors are responsible for triggering many SASP proteins, including IL-6, IL-8 and IL-1α, which intensify the SASP in a positive feedback manner. Although some SASP proteins recruit immune cells to eliminate senescent cells, others can escape from this clearance and express SASP proteins in a chronic manner to continue the inflammatory scenario and drive aging (for review see Rodier and Campisi, 2011). While many pathways mentioned in this review touch upon mechanisms that are common to neuronal failures as a result of elevated ROS and the mechanisms that play in age-related complications mentioned here, little is known about their specific relationships or how best to model such relationships experimentally. The age-related aspects that really should be recapitulated to provide tools for the modeling of late-onset diseases remain to be determined.

## Modeling diseases with iPSCs

Pathology in the CNS or “central” pathologies that are present from birth are often associated with clear and strong trends of heritability or specific *in utero* experiences. However, when the clinical onset of pathology occurs beyond the second decade of life, as frequently observed in PD, AD, and schizophrenia, tracing the underlying cellular mechanisms becomes even more complex. We decided to focus our discussion on these three pathologies due to the considerable insights gained through the use of stem cells with respect to both disease etiologies and the roles of oxidative stress, with the latter subject being a primary area of interest in our research group. Furthermore, all of these disorders share age-related phenotypes that are implicated in impaired levels of cognition. This suggests that the approach that propels neuronal aging *in vitro* will be particularly important for the modeling these disorders to enable the identification of age-related phenotypes, as it has already been shown for PD (Miller et al., 2013).

In this section, we have focused on ROS as the central player in the pathogenesis of these diseases as many age-related impairments ultimately converge on and negatively impact cell respiration, resulting in the overproduction of ROS.

### A special consideration of ROS

Due to recent advances in stem cell technology that now allow for the development of aged neurons to be integrated with genetic backdrops of adult neurological disease, we are uniquely positioned to conduct the *de novo* exploration of molecular networks that are common to aging, in addition to the co-existing pathological and redox states, such that new candidate targets may emerge that can harness oxidative stress and slow disease progression.

**Figure 1:**
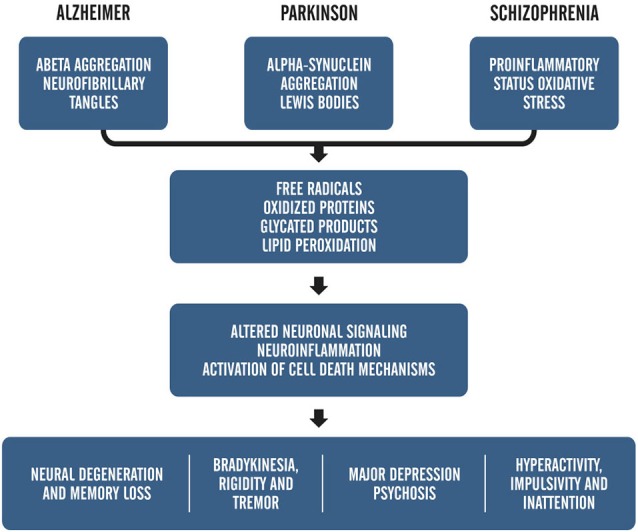
**Adapted from, “*ROS and Brain Disease: the Good, Bad and the Ugly*” Popa-Wagner et al., 2013**. This schematic shows the relationship between pathology-induced neuronal damage and ROS accumulation on overt clinical symptoms.

As mentioned before, besides being related to the aging process, ROS are also closely associated with the pathogenesis of several diseases, including AD, PD, and schizophrenia (Barnham et al., 2004; Uttara et al., 2009; Paulsen et al., 2012). Figure 1 provides an example of how the symptoms of oxidative stress and metabolic mismanagement may manifest upon the backdrop of disease and explains the alignment of these symptoms within known pathologies for prominent age-related neurological disorders.

**Figure 2:**
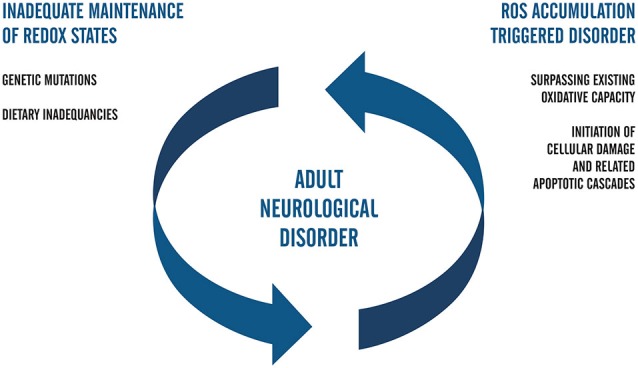
**Flow chart that represents the positive feedback model of ROS-related neuropathology in adults**. In this postulate, genetically inherited susceptibilities alone or those compounded by dietary inadequacies trigger the accumulation of ROS. Upon surpassing the redox capacity of the cell during the second phase of the cell cycle, ROS-related damage occurs and invokes death-signaling cascades. Positive feedback is complete when the biological capacity for redox management continues to fail to meet cellular needs and fails to adequately combat oxidative stress. Future studies regarding ROS in iPSC systems of neuropathology that aim to elucidate mechanisms of AD and PD may be facilitated by coupling the genetic background of accelerated aging with clinical factors in patients and carriers of early adulthood forms of neurological disease.

In this review, we interpret this form of disease modeling as one that captures the cycle of damage whereby the inherited disorder leads to cell damage and local elevations in ROS. In Figure 2, we have summarized what we understand to be key considerations in adult neurological disorders that would generate this type of positive feedback on ROS generation and ultimately generate a spiral of neurons towards cell death.

#### ROS in the interplay of neurodegenerative diseases

Mitochondrial dysfunction and oxidative stress operate together to produce the normal aging phenomenon. Therefore, it is unsurprising that these mechanisms could play important roles in the development of late-onset neurodegenerative diseases. Alterations in the redox system occur via genetic or non-genetic factors, which results in increased levels of ROS in most cases that trigger oxidative damage within the neural system. Here, we discuss some of the evidence that places ROS as a key molecule for late-onset neurodegenerative diseases. However, whether this event represents the cause or consequence of pathogenic processes is the focus of controversy. The major molecular alterations that occur in PD, AD, and schizophrenia are summarized in Table 1.

**Table 1:** **Summary of molecular cascades and involvement categorized by disorder**.

Disorder	Molecular changes affecting redox capacity	Age-related ROS imbalances
Parkinson’s disease	Parkin mutations, acts on β-catenin degradation	L-type Ca^2+^ channels in dopamine neurons increase intracellular calcium creating a metabolic stress with production of ROS
	α-Synuclein mutations, impairs proteasome-mediated proteolysis	
	Impaired mitochondrial complex I activity	PINK deficiency causes mitochondrial Ca^2+^ accumulation with stimulation of ROS production
	Reduced GSH activity	
	LRRK2 mutations (encodes the Ras/Rho-like GTPase domain)	
Alzheimer’s disease	Reduction in reelin	Disruption in calcium homeost­asis associated with increased ROS accumulation and damage
	Reduced Akt levels in Th neurons	
	Accumulation of reelin	p53 conformational alterations due to oxidative stress
	Aberrant GSK3β	
Schizophrenia	EPHB1 mutations, acts on GTPase (Ras/Rho)	Age-related reductions in Wnt signaling
	AKT-1, upregulated in oxidative stress	Increased ROS and extra mitochondrial O_2_ consumption
	Reduction in reelin	
	Reduced GSH activity	
	Impaired mitochondrial complex I activity	

##### Parkinson’s disease

PD is the second most common neurodegenerative disorder and most frequently affects elderly individuals. During the course of the pathogenesis of PD, the loss of dopaminergic neurons from the substantia nigra results in movement and postural dysfunction. With disease progression, neuronal loss spreads to other brain regions and symptoms such as hallucinations and dementia can emerge. Sporadic and familial cases share genetic components, although the etiology is unknown in most cases (For review see Bertram and Tanzi, 2005; Badger et al., 2014). Interestingly, oxidative stress appears as a common feature in patients. Reduced activity levels of complex I of the mt electron-transport chain have been described in patients with PD (Keeney et al., 2006). Furthermore, the selective inhibition of this complex by specific compounds such as rotenone can induce a parkinsonian phenotype (Cannon et al., 2009).

The genes that are most frequently associated with the pathology of PD are α-sinuclein (SNCA), leucine rich repeat kinase 2 (LRRK2), glucocerebrosidase (GBA), PTEN induced kinase 1 (PINK1), parkin (PARK2), and DJ (PARK7) (Badger et al., 2014). PINK1 and parkin function together, whereby PINK1 accumulates in the mt and parkin depolarizes the mt membrane to promote the autophagy of damaged mitochondria. Mutations in any of these genes lead to impaired functionality of the others, with consequent changes in mt metabolism. Dopaminergic neurons derived from iPSCs with PINK mutations are vulnerable to oxidative stress once the depolarization of mt membrane reduces the levels of glutathione synthase (GSH) and, as a consequence, increases ROS levels (Cooper et al., 2012). Mutations in LRRK2 of iPSCs-derived neurons leads to the accumulation of α-synuclein, the increased expression of oxidative stress genes, and vulnerability to hydrogen peroxide (Nguyen et al., 2011). Although the SNCA protein does not have a clear role in the pathology of PD, its accumulation leads to increased neuronal death due to their greater vulnerability to oxidative stress (Byers et al., 2011).

##### Alzheimer’s disease

As the most prevalent neurodegenerative disorder in elderly people, AD is characterized by the progressive loss of memory and cognitive decline (Bertram and Tanzi, 2005). The increase in life expectancy predicts that the prevalence of this disorder will rise considerably in future decades. The deposition of amyloid-β (Aβ) derived from the cleavage of amyloid precursor protein (APP) by β and γ-secretases and the hyperphosphorylation of Tau (a microtubule-associated protein) are the hallmarks of AD (Bertram and Tanzi, 2005; Jin et al., 2011). Oxidative damage occurs during the initial stage of AD (Nunomura et al., 2001) and leads to Aβ deposition and Tau phosphorylation (Melov et al., 2007). Moreover, superoxide dismutase (SOD) deficiency mice exhibit accelerated Aβ oligomerization (Murakami et al., 2011) and the hyperphosphorylation of Tau (Melov et al., 2007).

In addition to the well-described events that occur during the progression of AD, other molecular alterations also appear to be connected to the pathology. Patients with AD can exhibit the accumulation of reelin (Botella-López et al., 2006). The reelin protein is known to be involved with the organization of the developing CNS, whereas it has been implicated in signaling pathways implicated in neurodegeneration in the adult brain. During periods of elevated membrane potential, reelin typically interacts with the apolipoprotein E (ApoE) receptor to increase levels of intracellular calcium via the NMDA receptor (Herz and Chen, 2006). Therefore, changes in reelin signaling could potentially drive a mismatch between physiologically appropriate levels of calcium and the parallel activation of other cascades involved in neuronal excitation and the redox state (Bezprozvanny and Mattson, 2008).

In addition, a further two molecules have been also linked to the pathogenesis of AD: Akt and GSK3β. Akt regulates the functionality of GSK3β by inducing its phosphorylation, with the consequent inactivation of GSK. The so-called GSK3β hypothesis of AD has been explored (reviewed by Hooper et al., 2008) and the molecule is thought to be linked to the hyperphosphorylation of Tau and increased deposition of Aβ. Patients with AD frequently exhibit reduced levels of Akt (Ryder et al., 2004), which in turn increases the activity of GSK3β.

##### Schizophrenia

Schizophrenia is considered to be a heterogeneous chronic illness with a combined genetic background and environmental influences; the latter plays a fundamental role once the disorder occurs. In identical twins, schizophrenia develops in both twins in almost 50% of cases (Cardno and Gottesman, 2000). Defined as a neural connectivity disorder with regional synaptic deficits and abnormal synaptic pruning, it is believed to be a neurodevelopmental disorder that is commonly diagnosed in adolescence (Murray and Lewis, 1987; Insel, 2010). Apart from genetic changes, one biochemical factor that appears to be common between patients with schizophrenia is the increased levels of ROS that can impact many processes, including those in neurons. One hypothesis is that the largest source of ROS comes from complex 1 of the mt electron transport chain. It is interesting to note that platelets of patients with schizophrenia presents with higher complex 1 activity levels compared to healthy control individuals (Dror et al., 2002), which indicates that ROS production levels in patients with schizophrenia are high. Moreover, there are many other genetic and environmental risk factors for the development of schizophrenia that are known to induce oxidative stress (reviewed by Bitanihirwe and Woo, 2011).

Patients with schizophrenia tend to exhibit an older appearance than their biological age (Kirkpatrick et al., 2008). To date, the most widely accepted hypothesis for this observation was that the use of medication in combination with lifestyle factors resulted in this aged phenotype, with consequently reduced lifespans. Some groups have recently started to discuss whether inherited biological processes that underlie schizophrenia could contribute to the observed accelerate aging (Kirkpatrick et al., 2008; Fernandez-Egea et al., 2009; Tang et al., 2009; Jeste et al., 2011). Much biological evidence supports the theory that schizophrenia shares common events related to age-related disorders, with special consideration to the production of ROS.

During normal aging, oxidative stress levels increase while GSH activity decreases (Finkel and Holbrook, 2000; Liu et al., 2004; Currais and Maher, 2013). This is the first evidence that strengthens the theory that schizophrenia could be a syndrome of accelerated aging in which GSH activity is altered (Gysin et al., 2007). Moreover, reductions in telomere length, another classical hallmark of aging, have been observed in association with increased pulse pressures and a predisposition to diabetes in patients with schizophrenia, independent of the influence of medication (Fernandez-Egea et al., 2009). In terms of anatomical structures, some data has shown that the aging in brains of patients with schizophrenia accelerate faster than healthy subjects (Koutsouleris et al., 2014) with reductions in the integrity of white matter (Mori et al., 2007; Kochunov et al., 2013). In schizophrenia, oxidative damage can be detected in plasma even during the first episode of psychosis, where GSH has lower levels of activity (Micó et al., 2011); this could provide a useful diagnostic biomarker of the disease.

Additionally, changes in neuronal potential are often observed in patients with schizophrenia, which appears to be a consequence of oxidative stress (Ballesteros et al., 2013). This potential disturbance results from a malfunction of the NMDA receptors, another hallmark of schizophrenia. Disruption of NMDA receptors could occur due to the over-oxidation of extracellular sites of the receptor as a consequence of a deficiency in glutathione production (Steullet et al., 2006). Imbalances in the dopaminergic system are also frequently observed in schizophrenia, which may occur as a result of GSH alterations as reduced levels of activity results in decreased dopamine levels (Jacobsen et al., 2005). As ROS are products of the degradation of L-dopa and dopamine, the absence of GSH makes the substantia nigra vulnerable to toxic effects of ROS, with a consequent reduction in the number of dopaminergic neurons. Taken together, this data places ROS as a principle player in the molecular and cellular pathophysiological mechanisms underlying schizophrenia.

## Summary of critical targets for further consideration

In this review, we have summarized the numerous intersections between redox state signaling and disease-associated aberrant functions. Moreover, we have expanded our conceptual framework to include non-neuronal and potentially peripheral sources of ROS accumulation and aging cues in the development of late-onset neuropathologies such as PD, AD, or schizophrenia. Importantly, this review has emphasized those mechanisms that are likely to be causal to cognitive impairments, in addition to the roles of ROS in age-related neural damage. In Table 1, we have provided a reference tool of what we believe to be the molecular targets that can unravel the relationship between oxidative stress and late-onset neurological disorders. In most cases, the findings referenced below follow the development of detection protocols that could be readily adapted to use for the high-throughput screening of transformed neurons from iPSCs on the biological backdrops of pathology and age acceleration. Correspondingly, we predict great therapeutic gains from these loci in the years ahead.

The development of age-accelerated procedures that can convert stem cells into neurons may represent a powerful new opportunity to quantify relationships between genetic predisposition(s) or drug exposure(s) for any maturational or functional redirection that results in an otherwise determined biological system. Several strategies to accelerate aging have been explored in this review, including the overexpression of prelamin A/progerin to generate aged dopaminergic functional neurons, which were successfully derived from reprogrammed PD fibroblasts by Miller et al. The progerin disorders, however, overtly manifest symptoms throughout the body, but do not affect nervous system function. In order to explore alternative candidate pathways that might produce new models, we examined the literature for cascades that are altered by aging, which could propel the acceleration of aging in neurons to study late-onset forms of neuronal dysfunction. We have explored strategies based on studies related to brain aging and sought insights based on studies from other systems, which led us to suggest that adenosine and ROS may participate as potential inductors of aging *in vitro*.

Senescence in general is frequently associated with redox system deregulation, which directly impacts on ROS accumulation and oxidative stress levels. The pathologies discussed in this review are age-related and share common changes in ROS levels that may result from alterations in different metabolic pathways.

Going forward, an important goal in targeting and developing therapeutic interventions using neuronal microsystems of aged neurons is the extent to which the locus of the disorder is intrinsic vs. imposed upon the neuron. We encourage the modeling of late-onset diseases using iPSCs, but some details may need to be adjusted in order that such models could be used to study the causes of these diseases and novel pharmacological targets.

## Conflict of interest statement

The authors declare that the research was conducted in the absence of any commercial or financial relationships that could be construed as a potential conflict of interest.
